# Editorial: Improving quality of life in patients with differentiated thyroid cancer

**DOI:** 10.3389/fonc.2023.1154569

**Published:** 2023-02-20

**Authors:** Avi Hefetz Khafif, Oded Cohen, Gianlorenzo Dionigi

**Affiliations:** ^1^ Department of Otolaryngology, Soroka Medical Center, Ben Gurion University of the Negev, Beer-Sheva, Israel; ^2^ Department of Pathophysiology and Transplantation, University of Milan, Milan, Italy; ^3^ Division of Surgery, Istituto Auxologico Italiano, Istituto di Ricovero e Cura a Carattere Scientifico (IRCCS), Milan, Italy

**Keywords:** thyroid cancer, nerve monitoring, quality of life, thyroidectomy, neck dissection

Differentiated thyroid carcinoma (DTC) is on the rise worldwide and ranks first among endocrine cancers (1). The survival rate of DTC patients has increased in recent decades with the development of multidisciplinary screening and treatment methods (2). Thyroid cancer is most commonly diagnosed in women in their 40s, which means that these women live longer after completing treatment (3). The increase in thyroid cancer is mainly due to the discovery of thyroid microcarcinoma, and despite the increase in diagnoses, survival rates have not changed (4). This continued increase in new thyroid cancer cases and the known excellent prognosis for well-differentiated thyroid cancer have led to an increasing focus on the quality of life of thyroid cancer patients, rather than just complete removal of the tumour and adjuvant treatments.

Major efforts have been made to reduce the surgical burden in low-risk tumours and in diagnostic procedures. Other surgical contraventions, such as the role of central neck dissection, especially prophylactically, has been questioned given its risk-benefit ratio; the role of active survival in low-risk DTC; non-surgical interventions such as radiofrequency and thermal ablations are among the studies included in these current Research Topics. Other aspects relate to the use of technology to minimise potential complications and adverse outcomes of thyroid surgery - use of intraoperative nerve monitoring to reduce recurrent laryngeal nerve injury, remote surgical approaches that avoid visible neck scars, and more.

After completion of primary treatment, patients with DTC can usually remain healthy and return to their former lives (3, 4). However, in the transitional phase after completion of primary treatment, DTC patients may suffer varying degrees of long-term physical, social and psychological problems that make their survival much more difficult (2). Although some cancer-related problems diminish over time, some DTC patients struggle with physical (dysphonia, hypocalcaemia, pain, dysphagia), psychological (anxiety, depression, fear) and social (avoidance, re-employment) problems related to the treatment consequences (1–4). These problems affect the adaptability and quality of life of DTC patients learning to live with cancer and represent a major challenge in the recovery process (1) (**Figure 1**).

**Figure 1:**
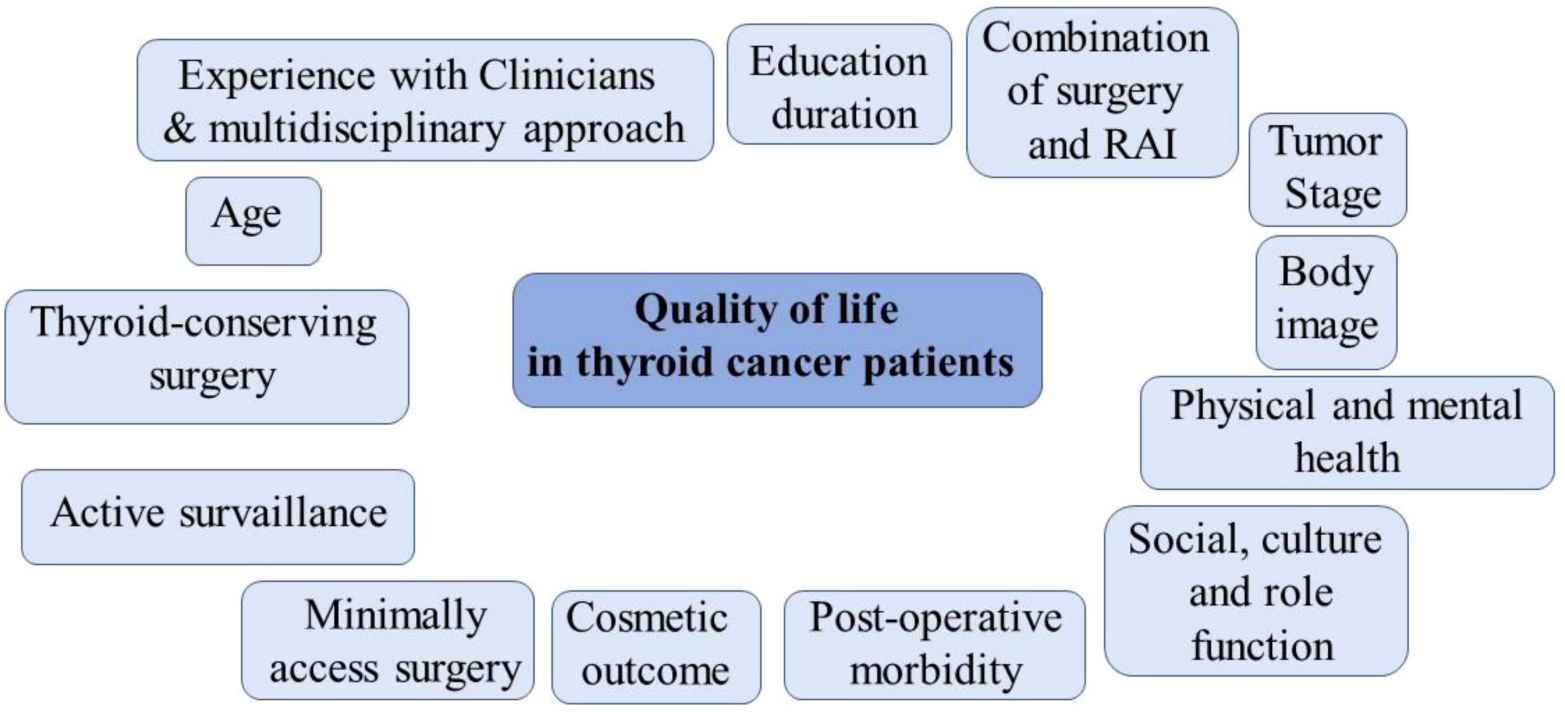
Factors influencing quality of life in thyroid cancer patients.

In addition to objective factors that have an obvious impact on quality of life, such as postoperative complications, recurrence, etc., subjective quality of life, i.e. patient-centred factors in treatment options for DTC, has become an important topic in recent clinical trials (1–4). This index distinguishes between general and disease-specific quality of life in terms of the spectrum of life domains affected. General quality of life should encompass all areas of life, while disease-specific quality of life focuses on the effects of disease and therapy (consequences).

Factors influencing the quality of life of DTC patients include physical and psychological symptoms, self-efficacy and social support (2, 3). Close counselling and education of DTC patients, care and symptom control have been shown to be particularly important (1–4). Particular attention needs to be paid to the early management of postoperative morbidity (1–4). Postoperative management has improved, but long-term data, especially on quality of life, are needed (5, 6).

The more severe the symptoms in DTC patients, the greater the psychological distress, the lower the physical and social functioning and the poorer the overall quality of life (7). Social support, such as feeling protected or receiving help from others, helps survivors to actively manage their health problems and find positive meaning in life, which ultimately improves their quality of life (1, 8, 9).

The concept of quality of life is currently gaining importance in the evaluation of treatment procedures (10). When comparing multiple treatments, standardisation is needed to account for the heterogeneity of patient cohorts. Analogous to relative survival in DTC epidemiology, the measured QoL scores should be set in relation to the age- and gender-specific reference of the general population to show the actual effect of the respective disease and its treatment.

Physicians should discuss the expected outcomes after thyroid surgery with patients in order to increase patient satisfaction and quality of life through detailed information.

## Author contributions

AK, OC and GD: writing and revising the manuscript. All authors contributed to the article and approved the submitted version.
